# Effects of biotic interactions on modeled species' distribution can be masked by environmental gradients

**DOI:** 10.1002/ece3.2657

**Published:** 2016-12-20

**Authors:** William Godsoe, Janet Franklin, F. Guillaume Blanchet

**Affiliations:** ^1^ Bio‐Protection Research CentreLincoln UniversityLincolnNew Zealand; ^2^ School of Geographical Sciences & Urban PlanningArizona State UniversityTempeAZUSA; ^3^ Department of Mathematics and StatisticsMcMaster UniversityHamiltonONCanada; ^4^ Département de BiologieFaculté des SciencesUniversité de SherbrookeSherbrookeQCCanada

**Keywords:** competition, dispersal, ecological niche, priority effect, range limits, species' distribution model

## Abstract

A fundamental goal of ecology is to understand the determinants of species' distributions (i.e., the set of locations where a species is present). Competition among species (i.e., interactions among species that harms each of the species involved) is common in nature and it would be tremendously useful to quantify its effects on species' distributions. An approach to studying the large‐scale effects of competition or other biotic interactions is to fit species' distributions models (SDMs) and assess the effect of competitors on the distribution and abundance of the species of interest. It is often difficult to validate the accuracy of this approach with available data. Here, we simulate virtual species that experience competition. In these simulated datasets, we can unambiguously identify the effects that competition has on a species' distribution. We then fit SDMs to the simulated datasets and test whether we can use the outputs of the SDMs to infer the true effect of competition in each simulated dataset. In our simulations, the abiotic environment influenced the effects of competition. Thus, our SDMs often inferred that the abiotic environment was a strong predictor of species abundance, even when the species' distribution was strongly affected by competition. The severity of this problem depended on whether the competitor excluded the focal species from highly suitable sites or marginally suitable sites. Our results highlight how correlations between biotic interactions and the abiotic environment make it difficult to infer the effects of competition using SDMs.

## 1 Introduction

**Figure 1:**
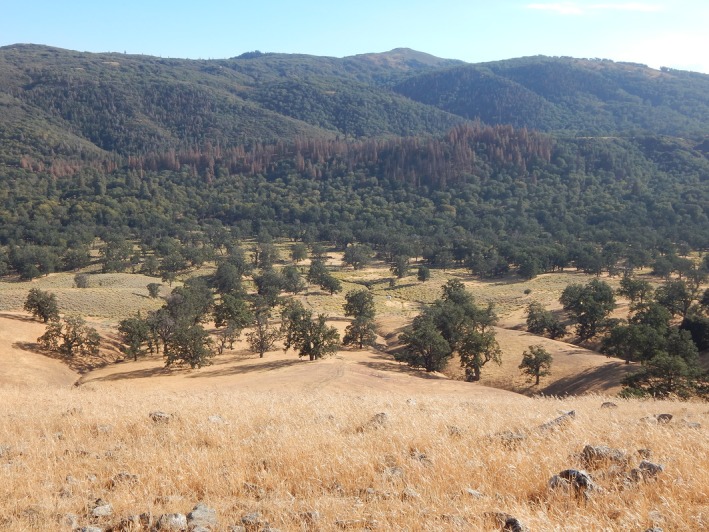
One of many systems where it is desirable to understand the impact of biotic interactions on species' distributions. Blue oak (*Quercus douglasii*) is the iconic species of mid‐elevation woodlands in California and is shown in the foreground dominating the equator‐facing slopes on the Tejon Ranch, Tehachapi Mountains, in southern California. Recent research shows that in field experiments, blue oak seedlings survive at high rates on more mesic poleward‐facing slopes and at higher elevations, shown in the background, and are probably excluded from the mesic part of the topoclimatic gradient by competition from the mixed conifer forest species that can be seen dominating those sites (Davis et al., 2016). This may change as climate change‐induced hot drought drives species‐specific tree mortality as seen in the photograph, changing the landscape of competition (photograph by J. Franklin, 30 September 2016)

A fundamental goal of ecology is to understand the determinants of species' geographic distributions (Chase & Leibold, 2003; May & MacArthur, 1972; Pulliam, 2000; Thuiller et al., 2013). We know that species' distributions depend on the joint effects of several factors, notably the abiotic environment, biotic interactions, and dispersal (Araujo & Guisan, 2006; Case, Holt, McPeek, & Keitt, 2005; MacArthur, 1972; Peterson et al., 2011). An increasing number of studies seek to understand the effects of biotic interactions on species' distributions (Figure 1; Thuiller et al., 2013; Wisz et al., 2013). Biotic interactions occur when one species alters the population growth rate of another species (Abrams, 1987). Prominent examples of biotic interactions include competition, predation, herbivory, host–parasite interactions, mutualism, or facilitation (Holland & DeAngelis, 2009). Biotic interactions are inherently complex and it is often unclear what information must be collected to identify their effects, although it has long been hoped that we can use information on species' distributions to detect the influence of biotic interactions (Diamond, 1975; MacArthur, 1972).

A potential approach to characterize species interactions is to modify a standard species' distribution models—hereafter species' distributions models (SDMs). For our purposes, SDMs are statistical models that seeks to estimate the probability of occurrence or abundance of a species of interest using independent variables describing the environments where the species could be observed (Elith & Franklin, 2013; Sexton, McIntyre, Angert, & Rice, 2009). Many SDMs include variables describing the abiotic environment such as precipitation and temperature and, to model biotic interactions, the occurrences or abundances of interacting species are also included as predictor variables in the model (Araújo & Luoto, 2007; Giannini, Chapman, Saraiva, Alves‐dos‐Santos, & Biesmeijer, 2013; Meentemeyer, Moody, & Franklin, 2001; Meier et al., 2010; Pellissier et al., 2012). Information about another species can be included as a predictor in any widely used SDM method (Franklin, 2010). A common SDM method to use for such analyses is generalized linear models (GLMs; Araújo, Marcondes‐Machado, & Costa, 2014; Guisan, Weiss, & Weiss, 1999; Meier et al., 2010; Pellissier et al., 2010). Many analyses using SDMs implicate biotic interactions in range limits (Sexton et al., 2009; Wisz et al., 2013; Zimmermann, Edwards, Graham, Pearman, & Svenning, 2010).

Although SDMs are used to identify the importance of biotic interactions or other ecological mechanisms (Elith & Leathwick, 2009; Fraterrigo, Wagner, & Warren, 2014; le Roux, Lenoir, Pellissier, Wisz, & Luoto, 2013; Roux, Pellissier, Wisz, & Luoto, 2014; Sexton et al., 2009), there are sound statistical reasons to be skeptical of this approach. In particular, abiotic environment often indirectly influences the effects of biotic interactions (Callaway et al., 2002; Davis, Jenkinson, Lawton, Shorrocks, & Wood, 1998; Sexton et al., 2009; Tylianakis, Didham, Bascompte, & Wardle, 2008) and the resultant correlations among variables (i.e., multicollinearity) can make it difficult to infer how important each variable is for shaping species' distributions (Graham, 2003). At present, the severity of this problem is unclear.

Our objective was to determine when we can identify the impact of biotic interactions using SDMs. To do this, we focused on a single type of species interaction, competition, because it is common in nature (Gurevitch, Morrow, Wallace, & Walsh, 1992) and its effects on species' distributions have been studied extensively (Araújo & Rozenfeld, 2014; Case et al., 2005; Godsoe, Murray, & Plank, 2015b; Pielou, 1974; Sexton et al., 2009; Soberón, 2010). To determine when SDMs can identify the effect of competition, we used simulations to create pairs of virtual species' distributions and then tested whether SDMs can accurately infer the known effects of competition. Our simulations were based on a model of competition (Hutchinson, 1978; Morin, 2009) where a species' success can depend strongly (but not entirely) on the abiotic environment. In some of our simulations, species' distributions were also influenced by dispersal among locations (Cantrell & Cosner, 1998) and/or priority effects (Fukami, 2015). We simulated 1,500 pairs of species where different strengths of competition, dispersal, responses to environmental gradients, and initial conditions were considered. We constructed SDMs to predict the abundance of one species using information about the abiotic environment and then tested whether the SDMs could be used to infer the importance of competition, by testing whether adding the abundance of the competitor as a covariate substantially improved the models. Because we have used a well‐understood model of biotic interactions, we posited that commonly applied SDMs would be able to infer the importance of competition. However, when the abiotic environment indirectly influences the outcome of competition, we hypothesized that SDMs may exaggerate the influence of the abiotic environment on species' distributions.

## 2 Materials and Methods

### 2.1 Simulation

**Figure 2:**
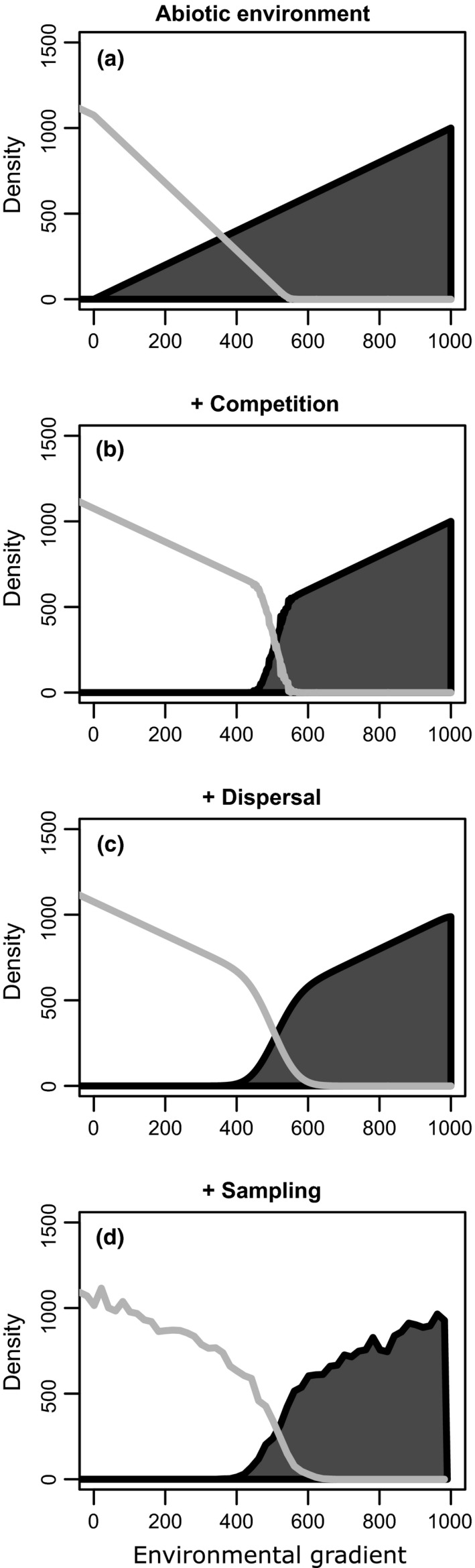
Example of changes in the density of the focal species (shaded region) and the competitor (light gray line) along a portion of the study region in one of our simulations. (a) In the absence of other mechanisms, each species' density gradually changes in response to the abiotic environment. The focal species' density increases with increasing values of *x*. Parameter values: *m*_1_ = 1, *m*_2_ = −2, *b*_1_ = 0, *b*_2_ = 1.075. (b) When competition is added to the simulation, each species may be eliminated from a portion of the study region. In this case, the focal species is eliminated when *x* is small, while the competitor is eliminated when *x* is large. Parameter values in addition to those used in panel (a): α_12_ = 0.75, α_21_ = 1. (c) Dispersal can allow each species to be present in habitats that would otherwise be unsuitable. Parameter values in addition to those used in panel (b): *D*_a_ = 10. (d) It is often impossible to know the density of each species in nature with absolute certainty, to reflect this; we simulate sampling of population densities at locations across the study region resulting in the jagged density lines in panel (d)

We simulated the effect of competition on two species each of which can occur across an environmental gradient. Figure 2 provides a four‐step overview of these simulations. As a first step, we generated an abiotic environmental variable *E* that can be measured across our study region and that changes as we move from one geographic location to another. Coordinates in geographic space are denoted with the symbol *x*. The carrying capacity of each species, defined as the population density that each species would reach at a given location in the absence of competition, changed along this gradient (Figure 2a). Second, we included competition between the two species. This can reduce the population density of each species or even eliminate each species from some locations (Figure 2b). In the third step, we added dispersal among locations, which smoothed the change in density of each species along the gradient (Figure 2c). Note that for some simulations, dispersal was not included among locations, in which case the species' distribution would resemble Figure 2b. In the fourth and final step, sampling uncertainty was included by adding noise to the species density observation (Figure 2d).

We used the Lotka–Volterra model to simulate species competing and dispersing across geographic space (Case et al., 2005; Pielou, 1974; Soetaert, Thomas, & Woodrow Setzer, 2010).

(1a) $$\frac{\partial n_{1}}{\partial t}=\overset{\mathrm{local}}{\overbrace{r_{1}n_{1}\left(1-\frac{n_{1}+\alpha_{12}n_{2}}{K_{1}}\right)}}+\overset{\mathrm{dispersal}}{\overbrace{D_{a}\frac{\partial^{2}n_{1}}{\partial x^{2}}}},$$

(1b) $$\frac{\partial n_{2}}{\partial t}=\underset{\mathrm{local}}{\underbrace{r_{2}n_{2}\left(1-\frac{n_{2}+\alpha_{21}n_{1}}{K_{2}}\right)}}+\underset{\mathrm{dispersal}}{\underbrace{D_{a}\frac{\partial^{2}n_{2}}{\partial x^{2}}}}.$$

This model describes the change in density of species 1 and 2 (*n*_1_ and *n*_2_, respectively). These densities can change across the spatial gradient *x* (which we simulated from *x *=* *−1,000 to *x *=* *1,000), or over time *t* (all our simulations were carried out for 1,000 time steps). Throughout this paper, species 1 will be considered the focal species and species 2 the competitor. The “local” terms in Equations (1a) and (1b) describe the ecology of the two species at a single location. The model assumes that locations are extremely small relative to the size of the study region; in practice, this means that individuals at a single location compete among each other at the same location, but not with individuals at other locations (Dieckmann, Law, & Metz, 2000). When the focal species is rare at a location where its competitor is absent, the focal species increased in density with an initial growth rate of *r*_1_. In the absence of competition, the focal species will increase in density until reaching its carrying capacity (*K*_1_). Increasing the density of the competitor reduced the population growth rate of the focal species. The term α_12_ describes the per capita harm that the competitor inflicts on the focal species. When α_12_ = 0, the competitor has no effect on the focal species. As α_12_ increased, the competitor progressively inflicts more harm to the focal species. Equivalent terms describe the change in density of the competitor at the same location. For example, *r*_2_ is the growth rate of the competitor when it is rare and in the absence of the focal species, just as *r*_1_ is the growth rate of the focal species. In our simulations however, we assume that *r*_1_ or *r*_2_ does not vary with *x* because the outcome of competition rarely depends on *r*_1_ or *r*_2_ (Case, 1999; Morin, 2009).

At a single location, our model can produce several different outcomes including competitive exclusion, where one species eliminates the other; stable coexistence, where the two species can survive at a single location indefinitely; or a priority effect (unstable coexistence), where either species can eliminate the other, depending on the initial densities of each species, which were specified at the start of each simulation.

We assumed that some individuals disperse passively among nearby locations. This is represented by the “dispersal” terms in Equations (1a) and (1b). Higher values of *D*_a_ indicate that individuals disperse more frequently.

As we move through space, the suitability of the environment can change (reflected by changes in the carrying capacity of the two species *K*_1_, *K*_2_). To describe these changes in the carrying capacity of the focal species, we use the equation:

(2) $$K_{1}=m_{1}x+b_{1},$$

where *m*_1_ describes how the carrying capacity of species 1 changes across the region (*m*_1_ is a slope). The parameter *b*_1_ represents the carrying capacity of species 1 when *x *=* *0 (in other words, *b*_1_ is an intercept). We define *x *=* *0 as the point where the focal species' carrying capacity is 0 and define *x *=* *1 as the point where the focal species' carrying capacity is 1 (Godsoe, Murray, & Plank, 2015a; Godsoe et al., 2015b). This sets *m*_1_ = 1, *b*_1_ = 0. Equation (2) was also used to define the carrying capacity of the competitor but with specific (and potentially different) values of *m*_2_ and *b*_2_ to define *K*_2_. After this simplification, our simulations used 25 combinations of parameter values for the competitor's response to the environment, *m*_2_ = −2, −0.975, 0.05, 1.075, 2.1, *b*_2_ = −2000, −975, 50, 1,075, 2,100.

We chose parameter values that allowed competition to produce several distinct effects including competitive exclusion and stable coexistence as well as unstable coexistence. We used five different values for the per capita effect of competition from the competitor on the focal species. In some simulations, the competitor had no effect on the focal species (α_12_ = 0), in others simulations competition was weak and stable coexistence was possible (α_12_ = 0.75), and in most of our simulations competition was strong (α_12_ = 1.5, 2.25, 3.00), making priority effects possible. We assumed that the strength of competition was constant across environmental gradients because the range limits often depend on the joint effects of *K*_1_ and α_12_. This makes it simpler to study range limits by investigating changes in carrying capacity (Godsoe et al., 2015b). As for dispersal, three strengths were simulated: no dispersal (*D*_a_ = 0), weak dispersal (*D*_a_ = 0.1), and strong dispersal (*D*_a_ = 10).

When coexistence is unstable, the outcome of competition depends strongly on the initial density of each species, and cannot be predicted by knowledge of the abiotic environment alone. This makes it necessary to specify the density of each species at the start of a simulation because the focal species can outcompete the competitor or vice versa, depending on the initial densities of each species. Given this ambiguity, we used four simulations for each set of parameter values with different initial conditions. In two simulations, the initial density of each species at each location was selected at random, from an exponential distribution with a mean of 0.01. In a third simulation, the focal species had a lower initial density than its competitor (0.01 vs. 1), and in a fourth simulation, the focal species had a higher initial density than the competitor (1 vs. 0.01).

We sampled the density of each species at one location every 10 units along the environmental gradient moving from *x *= −1,000 to *x *=* *1,000. This wide range of sampling ensured that many sites were outside of the fundamental niche of the focal species (a condition that we expect to be frequently met in empirical studies). However, most of our figures only depict samples from ~*x *=* *0 to *x *=* *1,000, the range of conditions where the focal species was present. To represent ecologist's uncertainty in the actual density of species found at a given site, we generated a random number from a Poisson distribution with a mean equal to the population density at this site (Figure 2d). We then measured an environmental predictor, denoted *E*, at each of these locations. *E* increases along geographic space, and *E* is measured without error. These observations were then used to generate SDMs (see Section 2.2). In our simulations, Species 1 was rarely present when *x *<* *0 because of our parameter choices. As a result, our illustrations of individual simulations such as Figure 2 start at *x *=* *0.

We used two measures for the importance of competition in our simulations, the *per capita* harm that the competitor inflicts on the focal species (α_12_; Equation 1a) and the percentage of sites where the focal species is absent because of competition. To determine this percentage, we counted the number of sites where the observed density of the focal species was greater than 0 when the competitor had no effect on the density of the focal species. We then computed the percentage of these where the focal species had a density greater than 0 when its competitor was present. We found that in the absence of competition, the focal species was present in a similar number of sites regardless of the strength of dispersal and the initial densities of either species. As a result, we did not account for these factors in this calculation.

In total, we simulated 1,500 datasets using all combinations of parameter values (see Table S1 for the list of parameter values explored). However, for 535 datasets, competition eliminated one of the species from the entire region. We chose to simulate all combinations of parameter values because there was no obvious way to systematically simulate the full range of possible species' distributions generated by our dataset while ensuring that both species were abundant. These datasets were ignored in subsequent analyses because it made little sense to estimate the effects of competition on a species' distribution when one of the competing species is absent. As such, 965 simulated datasets were considered for SDM analyses.

### 2.2 Species' distribution models

We applied SDM procedures recommended in Elith and Franklin (2013); Elith and Leathwick (2009); and Franklin (2010) to the abundance data generated by the simulations. Although it is common in the literature for SDMs to be fit to species presence–absence or presence‐only data using methods such as maximum entropy (Phillips, Anderson, & Schapire, 2006), when abundance data are available, modeling them with appropriate frameworks has been shown to improve SDM performance (Howard, Stephens, Pearce‐Higgins, Gregory, & Willis, 2014; Johnston et al., 2015). Abundance data have been used in studies of biotic interactions and SDM (Meentemeyer et al., 2001; Meier et al., 2010). The empirically estimated responses of a species to the environment can be linear (if only a portion of the gradient or species range is sampled) or complex (if indirect predictors are used, or if there are interactions among predictors) (Austin, 2002). For a preliminary set of datasets, scatterplots, regression trees (using the R package rpart (Therneau, Atkinson, & Ripley, 2014), and Poisson generalized additive models (GAMs) suitable for count response variable (using R function gam() in package mgcv (Wood, 2011), a spline smoother, and default dimension of the basis for the smoothing term), were used to visualize the relationship between the focal species (*n*_1_ or *n*_2_) and the environment *E* along with the competitor.

**Figure 3:**
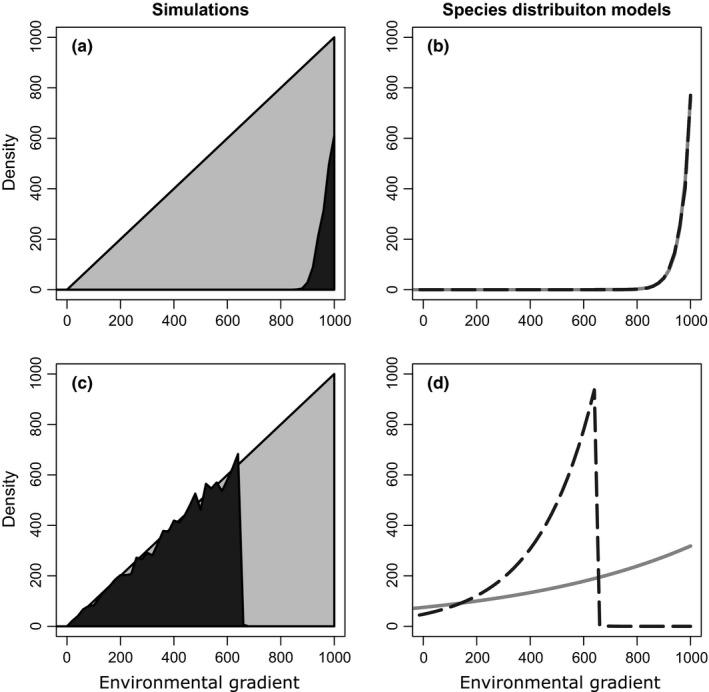
Species' distributions models (SDMs) had a mixed record of identifying the effect of competition on species' distributions in our simulations. Panels (a) and (c) plot the density of the focal species (gray shaded region) across the environmental gradient. In the absence of competition, the density of the focal species is linearly related to the environmental gradient. However, competition restricts the focal species to a subset of the gradient (black shaded region). Panels (b) and (d) show the density of the focal species predicted by two SDMs, one considering solely the abiotic environment (gray line) and the other considering both the abiotic environment and competition (black dotted line). In panel (b), the two SDMs made predictions that are almost indistinguishable and it is difficult to identify the effect of competition. In contrast, panel (d) shows that an SDM including species interactions (black dotted line) accurately describes the abundance of the focal species, while an SDM including only the abiotic environment does not (gray solid line)

Because the exploratory analyses suggested that the relationship between the focal species and the environment and the competitor were linear, we used GLMs, to estimate the impact the environment and the competitor had on the focal species. This approach is particularly useful for examining the importance of biotic interactions as the resulting parameter estimates are easy to interpret (Pellissier et al., 2010). For other purposes, notably, prediction it might be more appropriate to use more complex nonlinear models (Elith & Leathwick, 2009). Quasi‐Poisson models were used to account for the over dispersion observed in these datasets (Ver Hoef & Boveng, 2007). A zero‐inflated Poisson model would address the excess of sites with densities of 0 in some of the datasets (Barry & Welsh, 2002; Martin et al., 2005; Wenger & Freeman, 2008); however, zero‐inflated Poisson models could not be estimated because for some simulated data, there were abrupt boundary between presence and absence of individual for the species (Figure 3c), making it difficult to identify unique parameter estimates.

For each simulated “community,” we fitted two SDMs using the R statistical programming environment (R_Core_Team 2016), one ignoring the competitor and one including it:

Model ignoring the competitor: 
glm(formula = n1 ~ E, family = quasipoisson)



Model including the competitor: 
glm(formula = n1 ~ E + n2, family = quasipoisson)



Here, n1 is the density of the focal species, n2 is the density of the competitor, and E is a predictor (explanatory variable) describing the environmental conditions at a given location. To study the effect of competition in the SDM, we used the proportion of deviance explained by the SDM weighted by residual degrees of freedom. This statistic has been called *D* ^2^ by Guisan and Zimmermann (2000). This statistic is analogous to the coefficient of determination for a linear model. We reported three different measures for each model: *D* ^2^ with only environment as a predictor, the improvement (change) in *D* ^2^ when the competitor is included, in addition to the environment, and the regression coefficient (β_competitor_) associated with the other species, which is an estimate of how the density of the focal species changes when the density of the competitor increases. We selected these two statistics because the amount of deviance explained and parameter estimates are two common ways to evaluate the importance of a variable in regression models (Crawley, 2005). Our approach seeks to emulate typical statistical analyses of species' distribution conducted at a landscape scale (Giannini et al., 2013; Leathwick, 2002).

Of the 965 simulated datasets on which SDMs were calculated, it sometimes happened that one of the species was rare enough to interfere with model fitting. As a result, we have information on *D* ^2^ for 886 of the datasets and an estimate of β_competitor_ for 721 of the simulated datasets.

### 2.3 How effective were the SDMs at identifying the effect of competition?

If SDMs can identify the effect of competition on species' distributions, then simulations where the competitor excluded the focal species from many locations should result in SDMs where the *D* ^2^ attributed to the environment is small. In these simulations, a great deal of deviance should be attributed to the density of the competitor. These same simulations should show negative values of β_competitor_, indicating that the SDM predicts that the density of the focal species is lower in locations where the density of the competitor is high.

**Figure 4:**
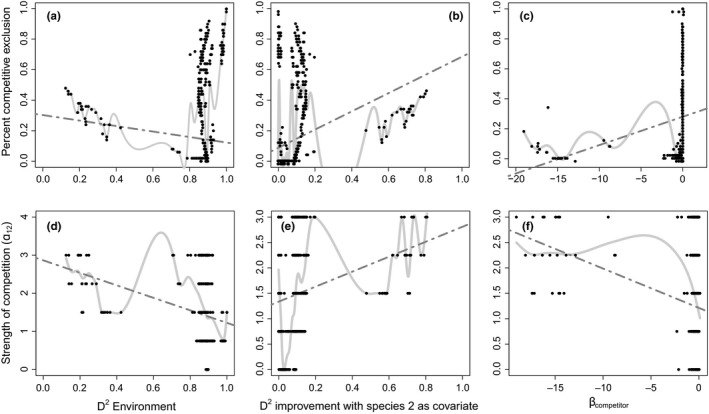
Scatterplots of the effect of competition on the distribution of the focal species for each of the statistics derived from species' distributions models (SDMs) (columns) and each measure of the importance of competition (rows). Each black point represents a single simulation; the dark gray dashed line represents the relationship inferred by a linear model and the light gray solid line represents the relationship inferred by a generalized additive model. a) percent competitive exclusion versus D^2^ Environment, b) percent competitive exclusion versus D^2^ improvement with species 2 as a covariate c) percent competitive exclusion versus β_competitor_. D) strength of competition (α_12_) versus D^2^ environment e) Strength of competition versus D^2^_improvement_ with species 2 as covariate f) Strength of competition versus β_competitor_

We are most concerned with how useful the outputs of SDMs can be as an index showing the importance of competition (for our purpose, it is either the per capita harm that a competitor inflicts on the focal species or the percentage of sites where the focal species is absent because of competition). An index used to infer the importance of competition should ideally be linear so that a change in the output of the SDMs can easily be interpreted as a change in the importance of competition (Figure 4). As such, we present coefficients of determination (*R* ^2^) from linear models for the relationships between the importance of competition and SDM parameters. This is a measure of how much of the variability in the importance of competition can be explained by the SDM output. In our case, a *R* ^2^ of 1 indicates that all of the variability in the importance of competition can be explained using output from SDMs, while a value of, say, 0.01 indicates that only a small amount of the variability in the importance of competition (1%) can be explained by the output of the SDMs. Following accepted usage, we do not assume a priori that the relationships in our study are linear; instead, we ask how much variability can be attributed to a linear relationship (Chesson, 2000; MacArthur, 1972; Rice, 2004).

To determine whether it would be easier to summarize information from this analysis using nonlinear relationships, we also fit Gaussian local‐scale additive models, which have two link functions, one for the means and one for the standard deviations of the models. The identity link function was used for the models' mean, while a log link function (Equation (3)) was used for the models' standard deviation (σ):

(3) log(σ−b),

where *b* is a parameter akin to an intercept term in a regression ensuring that the value of the standard deviation after applying the link function does not become zero, which would cause singularity problems when calculating likelihoods. In this analysis, *b* was fixed to 0.01. This type of additive model allows for the smoothed estimate to changes in the mean and standard deviation of the model. We present expected degrees of freedom (EDF, a description of the complexity of each smoother) for both the mean and standard deviation. These models were constructed using the gam() function in the mgcv package in R.

To relax the assumption of linearity, we investigated how often information derived from SDMs agreed with the true effect of competition. More specifically, we determined the probability that in a randomly selected pair of simulations, information from SDMs would lead us to believe (incorrectly) that competition is more important in one simulation when it is actually more important in the other. This probability is an alternative summary of the information in Kendall's tau (a nonparametric correlation coefficient; see Appendix S1).

## 3 Results

The relationship between the importance of biotic interactions in our simulations and the importance of biotic interactions inferred by SDMs was highly nonlinear, and on occasion highly variable (Figure 4). Thus, it would be difficult to reliably determine the importance of competition using SDM outputs (Table 1).

**Table 1:** Ability to infer the importance of competition from species' distributions models (SDMs)

Statistic derived from SDM	*R* ^2^ from linear model	Probability of ranking incorrectly^a^	τ	Mean estimated degrees of freedom^b^	σ Estimated degrees of freedom
Percent competitive exclusion					
$D_{\mathrm{environment}}^{2}$	.007	.389	−0.22	29.41	8.153
$D_{\mathrm{improvement}}^{2}$	.086	.267	0.47	36.5	8.45
β_competitor_	.021	.331	−0.17	7.91	8.1
α_12_					
$D_{\mathrm{environment}}^{2}$	.003	.441	−0.12	17.26	7.37
$D_{\mathrm{improvement}}^{2}$	.027	.504	−0.01	24.34	7.275
β_competitor_	.040	.331	−0.33	4.51	1.01

Tau refers to Kendall's tau, while estimated degrees of freedom are derived from Gaussian local‐scale additive models.

a As we describe in our Appendix S1, the probability of that two rankings disagree for a pair of observations is another way to represent the information in Kendall's tau.

b The mgcv package recommends checking dimension of the basis vector. In each case, we checked this and typically set this parameter to 4. In one case, a lower limit was set for computation 9 to speed up computation, and in another, a higher limit 100 was set.

Figure 4 presents pairwise scatterplots of each measure of the importance of competition and the output from SDMs. In each panel, the relationship between the importance of competition and the output of the SDMs was complex and variable. As a result, *R* ^2^ values from the linear model (dark gray dashed lines) were typically low (<.09), indicating that only a small amount of the variability in the importance of competition could be predicted using summary statistics derived from SDMs (Table 1). There was a high probability of incorrectly ranking the dataset in which competition is most important (Table 1). The Gaussian local‐scale additive models indicated a complex relationship between the importance of competition and the statistics derived from SDMs (Table 1; light gray solid lines on Figure 4). It is possible that more combinations of parameter values would be needed to produce SDMs with intermediate *D* ^2^ values.

The ability of SDMs to detect competition depended on whether the competitor excluded the focal species from locations that were marginally suitable to the focal species or sites that were most suitable. Figure 3a shows a simulated dataset where the SDM was misleading. In this simulation, competition eliminated the focal species from sites that were marginally suitable (i.e., sites where the focal species' carrying capacity was low), while SDMs attributed a great deal of deviance to the abiotic environment. Adding information on the abundance of the competitor did little to improve the model (Figure 3b). This problem occurred in many simulated datasets that resulted from a range of parameter combinations. These simulations populate the upper right corner of Figure 4a and the upper left corner of Figure 4b. In Figure S1, we illustrate the distribution of the focal species in 25 simulated datasets with similar problems.

Figure 3c shows a simulated dataset where it was easier to infer the importance of competition using an SDM. In this dataset, the focal species is excluded from sites where its carrying capacity was highest. In this case, an SDM including competition offered better predictions than an SDM ignoring competition (Figure 3d). In all the other simulations where a small amount of deviance was attributed to the abiotic environment and a large amount of deviance was attributed to the abundance of the competitor, the competitor excluded the focal species from sites where the focal species' carrying capacity was highest. These are illustrated in Figure S2.

Simulations where the estimated effect of the competitor on the focal species was unusually strong (β_competitor_ was strongly negative) tended to be those where the focal species and its competitor rarely encountered one another. These were often simulations where the competitor had a small effect on the distribution of the focal species (Figure S3). As such, values of β_competitor_ dramatically lower than zero were a poor guide for the importance of competition.

## 4 Discussion

There is a natural inclination to interpret SDMs as providing insights about the mechanisms shaping species' distributions (Alvarez‐Martínez, Suárez‐Seoane, Palacín, Sanz, & Alonso, 2015; Fraterrigo et al., 2014). Our results suggest that it is difficult to identify the effect of competition on species' distributions using SDMs under some circumstances, or to distinguish the effects of competition from other drivers. In many of our simulations, a great deal of variability was attributed to the abiotic environment. Including a competitor as a covariate produced modest improvement in our SDMs, regardless of the importance of competition. Below, we discuss the generality and implications of these results.

A key insight of our work is that the distribution of a species can appear to be determined primarily by the abiotic environment even when competition has a strong influence on where it is found. This is because the abiotic environment influenced the success of each species at each location. The resulting indirect effects of the environment mediate the suitability of a location for the competitor, which in turn influenced the presence or density of the focal species. In other words, in our simulations there was a strong correlation (multicolinearity) between the effects of the abiotic environment and the effects of competition, and multicollinearity is well known to affect variable selection and prediction in SDMs (Dormann et al., 2013). We even observed this correlation in simulations where the outcome of competition depended on the initial density of the two competitors (i.e., simulations where priority effect makes it possible to predict which species succeeds at some locations). From our simulations, we were only able to detect the effect of competition in cases where the competitor was abundant (the environment was suitable), the effect of the competitor on the focal species was strong, and the competitor excluded the focal species from abiotic environments that seemed most suitable to the focal species. For example, in Figure 3c, the focal species abundance increased along the environmental gradient in the absence of the competitor, but the focal species was entirely absent at high values of the environmental gradient when the competitor was present.

For many of our simulations, the effects of competition were difficult to detect using SDMs. This problem was particularly striking when the focal species was so rare that it was difficult to fit SDMs, but substantial problems occurred, even when both competitors were common. Figure 3a and b illustrates these cases where we never observed the focal species in the absence of the competitor and so we had no way to contrast the effects of biotic interactions and the abiotic environment on the focal species. Figure S3 shows 20 other simulated datasets where competition strongly influenced the distribution of the focal species, but SDMs attributed a great deal of deviance to the abiotic environment. In our simulations, the best guide to the utility of SDMs was whether the competitor excluded the focal species from marginally suitable locations or locations where the focal species' carrying capacity was highest.

We emphasize that our simulations were designed to systematically explore different qualitative outcomes of competition; they cannot be interpreted as representing common outcomes of competition in nature. Our results showed that it could be difficult or impossible to infer the importance of competition using SDMs. We showed that problems could occur with or without dispersal, when the two species coexist stably at some locations or whether a priority effect allows each species to exclude the other from the same location. But we do not know how commonly these conditions are found in nature. In other words, the larger number of simulations (combinations of parameters) for which it was difficult to detect competition effects is not necessarily proportional to the kinds of competitive interactions found in nature. In cases like Figure 3c, the presence of a competitor produces an abrupt range limit that can implicate competition. In Figure 3c, we can contrast the response of the species of interest across an abrupt change in density between similar abiotic environments where its competitor is present and absent. However, a more complex SDM such as a GAM or boosted regression tree might estimate the response of the focal species to the abiotic environment as a nonlinear function, erasing the signature of biotic interactions. This makes it important to use an understanding of the natural history of the study systems to identify potential cases where biotic interactions might be influencing species abundance or range limits (Giannini et al., 2013; Leathwick, 2002; Wisz et al., 2013). Particularly, there are mechanisms other than interactions among species that produce abrupt boundaries in a species' abundance (Abrams, 2009). Dispersal barriers may also generate natural experiments if they keep a competitor from reaching some regions where the focal species is present. When this is the case, we can contrast the distribution of the focal species when its competitor is present and when it is absent. For example, Anderson and Peterson (2002) studied the distributions of two species of pocket mice (*Heteromys australis* and *Heteromys anomalus*) and found that only one species occurred in some regions while both species occurred together in other regions. The authors could contrast the distributions of each species across regions to infer the effect of competition.

Depending on the application, the challenges of empirically identifying an effect of competition on species' distributions may represent either a modest caveat or a severe limitation on the usefulness of SDMs to study biotic interactions. When using SDMs to describe where a species is present (interpolation sensu Franklin, 2010), it may be acceptable to fit SDMs that attribute a great deal of variability to the abiotic environment, even when competition is important. When using SDMs to predict a species' distribution in a new time or location (Elith, Kearney, & Phillips, 2010; Franklin, 2010), a misspecification of the role of competition might lead to misleading extrapolations (Davis et al., 1998; Godsoe et al., 2015b).

It remains an open question how prevalent these indirect effects of environment are; we suspect they are common. Previous reviews have considered this phenomenon (Sexton et al., 2009; Wiens, 2011), but it is difficult to do the large‐scale manipulative experiments that would be needed to identify these indirect effects in nature (Narwani, Alexandrou, Oakley, Carroll, & Cardinale, 2013). Both Holt and Barfield (2009) and Godsoe and Harmon (2012) showed indirect effects of the abiotic environment in mechanistic models of consumer resource dynamics. Case et al. (2005) illustrated several models where environment and competition jointly shaped species' distributions, including metapopulation models with dispersal among locations. Some empirical studies and simulations show that biotic interactions become less important at large spatial scales.(Araújo & Rozenfeld, 2014; Fraterrigo et al., 2014; Soberón, 2010), although it has recently been pointed out that these results implicitly assume that regional coexistence mechanisms are strong, an assumption that is often not met in nature (Godsoe et al., 2015a).

The simple Lotka–Volterra models we used to provide a useful starting point for understanding how indirect effects of environment on competition might alter our interpretation of SDMs. However, Lotka–Volterra models have several idiosyncrasies that limit the generality of their results. In experiments, more complex models were required to predict the equilibrium density that each species will reach in the presence of a competitor (Ayala, Gilpin, & Ehrenfeld, 1973; Loreau, 2004). For the sake of simplicity, we only investigate pairwise interactions here, although more complex dynamical behavior can emerge with multispecies competition. For an illustration of range limits under multispecies competition, see Mohd, Murray, Plank, and Godsoe (2016). In more complex models such as the “Lottery model” (Chesson & Warner, 1981), spatial and temporal variability can have counterintuitive effects on the outcome of competition, which in turn might reduce the large‐scale effects of competition. Our model makes the strong assumption that competition only occurs within each location and that each location is small relative to the size of study region (Law, Murrell, & Dieckmann, 2003). Different behavior emerges from spatially explicit models where species can compete across adjacent locations (Bolker, Pacala, & Neuhauser, 2003; Chesson, Donahue, Melbourne, & Sears, 2005; Dieckmann et al., 2000; Snyder & Chesson, 2004). Although there is intuitive appeal to models explicitly designed to capture species interactions across spatial scales, competition among organisms often occurs at a fine spatial scale (say over a few meters), which is dwarfed by the scales of studies carried out by biogeographers (Soberón, 2007, 2010).

Our simulations were carried out across one spatial dimension (as if we have a latitudinal gradient but ignore longitude). We believe that this simplification is appropriate even though simulations in one dimension sometimes miss outcomes of competition that emerge in two spatial dimensions. Two‐dimensional models of competition produce different dynamics when dispersal and competition interact in complex ways (Dieckmann et al., 2000; Nowak, 2006). But in our simulations, these phenomena are less likely to occur because competition happens at an extremely fine spatial scale relative to the study region and dispersal is modest across the gradient. Case et al. (2005) present a model analogous to ours and illustrate simulations in both one and two spatial dimensions.

In our simulations, we assumed that the carrying capacities of each species changed linearly across the study region. If the changes in carrying capacity were nonlinear, additional complications could emerge. For example, it would be possible for the focal species to persist at the edges of the study region (high and low values of *x*), even though competition eliminates the focal species from the center of the gradient (intermediate values of *x*; see figure 1 in Austin, 2002).

In some cases, it may be possible to use SDMs to infer the importance of competition when additional information is available. If two species have a response to a single gradient, this may mask the effect of their competition, but SDMs typically include multiple environmental predictors, invoking the concept of the Hutchinsonian hypervolume (Elith & Franklin, 2013). An additional environmental gradient that has a strong effect on the focal species but not the competitor might allow the effect of competition to be detected if enough observations are available to overcome multicollinearity, model misspecification, and the curse of dimensionality (Hastie & Tibshirani, 1990). On the other hand, multiple species making up communities interact in many ways, some of which will affect their distributions, making it unlikely that biotic interactions can be recovered from distribution data alone. Where community composition is known, community distribution models (Clark, Gelfand, Woodall, & Zhu, 2014; Ferrier, Manion, Elith, & Richardson, 2007) may in some cases be able to use species co‐occurrence data to infer biotic interactions (Ovaskainen, Hottola, & Juha, 2010).

Inferences regarding the effect of competition on species' distributions may also require multiple lines of evidence from natural and designed information on species' traits, experiments, and phylogenies (Pigot & Tobias, 2013). Simulated data are part of the toolbox available for checking the assumptions of other approaches.

## Conflict of Interest

None declared.
