# Supplementary material for: Effects of biotic interactions on modeled species' distribution can be masked by environmental gradients
Source: Ecol Evol. 2016 Dec 20;7(2):654–64. doi: 10.1002/ece3.2657 (PMC5243196; doi:10.1002/ece3.2657)
Supplement: Supplementary file 1 [file ECE3-7-654-s001.docx]

Online Appendix 1: Supplemental materials.

Table S1: List of parameter values used in our model.

| Parameter | Values simulated | | | | |
| --- | --- | --- | --- | --- | --- |
| *D_a_* | 0 | 0.1 | 10 |  |  |
| $\alpha_{12}$ | 0 | 0.75 | 1.5 | 2.25 | 3 |
| *m*_2_ | −2 | −0.975 | 0.05 | 1.075 | 2.1 |
| *b*_2_ | −2000 | −975 | 50 | 1075 | 2100 |
| Densities at beginning of simulation | *n*_1_ and *n*_2_ at each site chosen at random | *n*_1_ and *n*_2_ at each site chosen at random | *n*_1_=0.1  *n*_2_ =1 | *n*_1_=1  *n*_2_ =0.01 |  |

**Connection between Kendall’s Tau and the probability of misclassifying a pair of observations.**

Kendall’s tau describes how often two measurements agree for a randomly selected pair of observations in a dataset. Pairs that agree are called concordant. The number of pairs that agree is denoted *C*. Pairs that disagree are called discordant. There are *D* such pairs. Assuming no ties, the total number of pairs is *N* such that:

*N=C+D* (1)

Kendall’s (τ)is typically defined as the difference between the portion of pairs that are concordant and the portion of pairs that are discordant:

*τ=(C-D)/N* (2)

In a given dataset, probability that a pair of observations is concordant (*c*), and the probability that a pair of observations is discordant is (*d*). These can be found as:

*c=C/N* (3a)

*d=D/N* (3b)

Substituting Equation 3a and 3b into Equation 1, we find that:

*c+d*=1 (4)

If we know τ, we can find *c* and *d*. Substituting equations 3a and 3b into equation 2 gives:

τ=*c-d*. (5)

Re-arranging equation (4) we find that

*c*=1-d

this can be substituted into equation (5)

giving:

τ=1-*d-d*

re-arranging we find that

*d*=-( τ-1)/2

hence, once we know τ we can find *d* the probability that a pair of observations will be discordant.


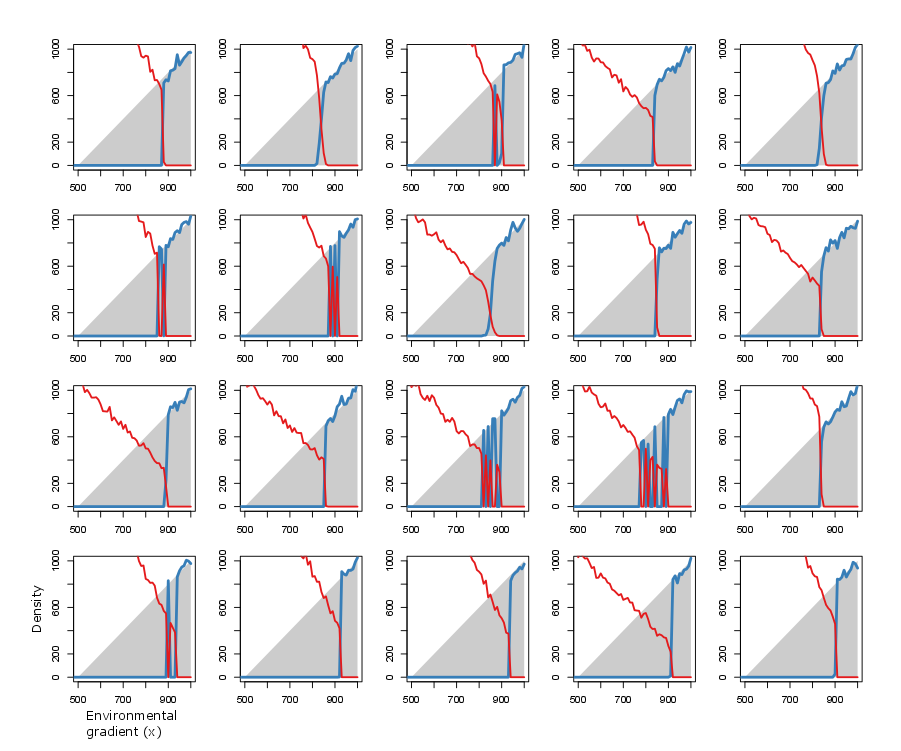


Figure S1: Illustrations of selected simulated species’ distributions where competition strongly influenced the distribution of the focal species (% competitive exclusion is between 60% and 99%), but SDMs strongly implicated the abiotic environment *D^2^_environment_*>0.9. Each panel is a plot of observed species density versus position along the environmental gradient. The blue line is the observed density of the focal species, the red line is the observed density of the competitor and the grey triangle represents the density of the focal species in the absence of the competitor.


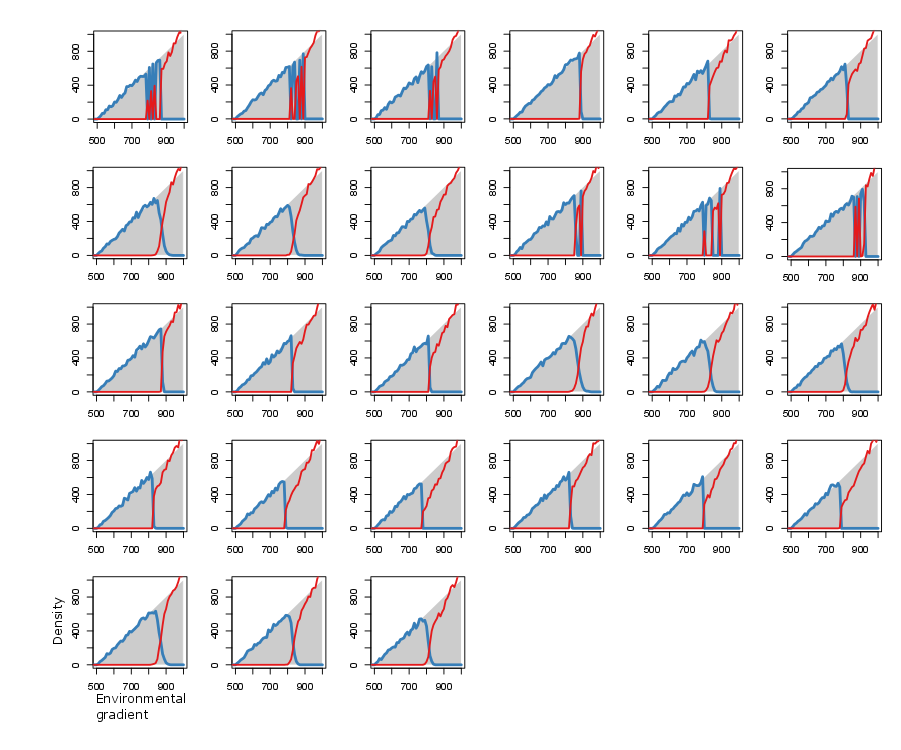


Figure S2: Illustrations of the 27 simulated species’ distributions where SDMs attributed a small amount of variability to the abiotic environment. Each panel is a plot of the observed population density versus position on the environmental gradient (compare to Figure 3 A, C in the main text though the environmental gradient is labeled by site rather than position on *x*). Here the observed density of the focal species is blue, while the observed density of the competitor is red. The grey triangle represents the population density of the focal species in the absence of competition. In each of the 27 simulations the competitor excludes the focal species from the right most portion of the graph while the focal species excludes the competitor towards the center of the graph.


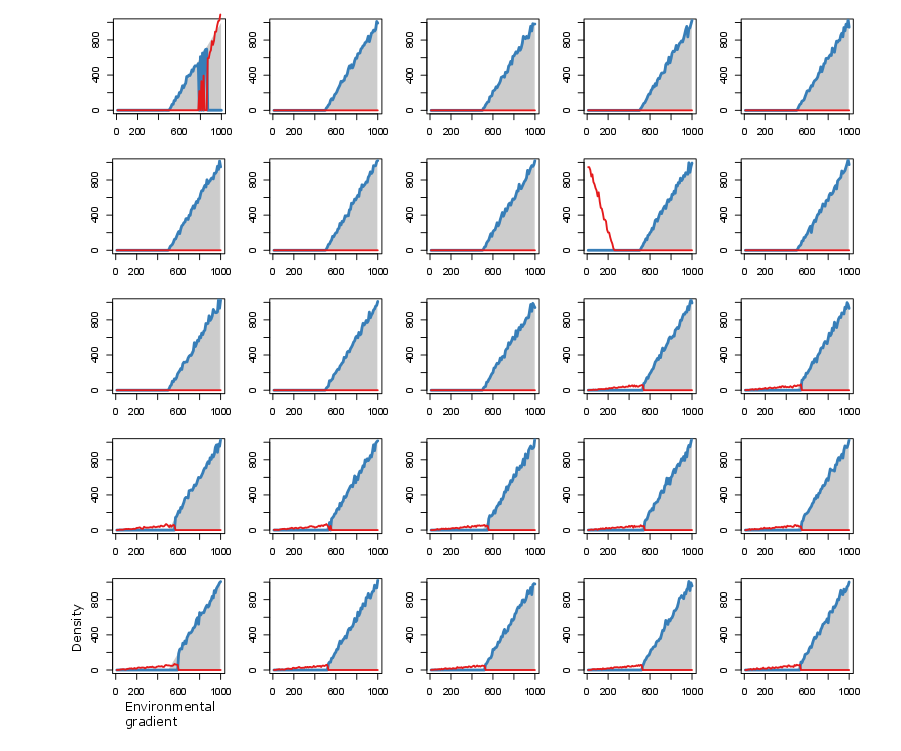


Figure S3: Illustrations of 25 simulated species’ distributions where *β_competitor_* < -5, indicating simulations where SDMs predict that the abundance of the focal species decreases dramatically as the abundance of the competitor increases. Each panel is a plot of observed species density versus position along the environmental gradient. The blue line is the observed density of the focal species; the red line is the observed density of the competitor and the grey triangle represents the density of the focal species in the absence of the competitor.
